# Advances in Alcoholic Liver Disease

**DOI:** 10.1155/2012/563018

**Published:** 2012-04-04

**Authors:** Natalia Osna, Kusum Kharbanda, Laura Schrum, Angela Dolganiuc

**Affiliations:** ^1^Department Internal Medicine, Omaha VA Medical Center, University of Nebraska Medical Center and Liver Study Unit, Omaha, NE 68198, USA; ^2^Liver Study Unit, Omaha VA Medical Center, Omaha, NE 68105, USA; ^3^Department of Internal Medicine, The Liver-Biliary-Pancreatic Center, Cannon Research Center, Carolinas Medical Center, Charlotte, NC 28203, USA; ^4^Department of Medicine, University of Florida, Gainesville, FL 32611, USA

This special issue reflects on multiple factors/mechanisms involved in the pathogenesis of ALD. Alcoholic liver injury is known to cause a broad range of liver abnormalities. Alcohol is primarily metabolized in the hepatocyte leading to increased secretion of inflammatory mediators, which, in turn, activate and/or influence the response of the nonparenchymal cells (NPCs) (hepatic stellate cells, Kupffer cells, and sinusoidal endothelial cells) and subsequently control the degree of liver injury.

This issue overviews general aspects of ALD, such as oxidative stress and inflammation (A. Ambade and P. Mandrekar) as well as the molecular aspects of these events, including the role of ethanol-metabolizing enzymes, ADH (T. Haseba et al.), and CYP2E1 (A. I. Cederbaum et al.) in the development of alcohol-induced liver injury. In addition, signaling mechanisms induced by alcohol are examined in the paper by L. N. Gerjevic et al. As a separate pathogenic aspect, the role of microRNA in ALD is analyzed in the paper by S. Bala and G. Szabo.

The consequences of alcohol-related liver injury, such as impairment of receptor-mediated endocytosis and lipid droplet accumulation, are presented in experimental *in vitro* studies of C. A. Casey et al. and B. McVicker et al., respectively.

One of the mechanisms that affects various liver cell types and affect disease progression is impairment of methylation reactions. In our special issue, the role of impaired methylation in pathogenesis of steatohepatitis as well as treatment modalities with promethylating agent, betaine, is discussed in the paper by C. H. Halsted and V. Medici and K. Kharbanda et al.

Liver also serves as an immune organ and accommodates a wide variety of cells, including immune cells. The latter consists of dendritic cells (DCs), natural killer (NK) cells, and lymphocytes, which are present in normal livers. Selective recruitment and retention of certain immune populations occurs during diverse liver diseases, and these cells play a critical role in the development and resolution of liver inflammation, remodeling, and destruction and actively participate in immune defense. The role of adaptive immunity in ALD development is overviewed by E. Albano. Also, the role of stem cells in ALD treatment is discussed in the paper by M. Pai et al. Finally, to underline the role of alcohol in progression of chronic infections (HCV), we included the paper by M. Neuman et al. which reflects on the markers of inflammation and fibrosis in alcoholic hepatitis and hepatitis C.

Each paper received external blind review in addition to our reviews.

This special issue covers exciting new areas of ALD pathogenesis and treatment and is strongly recommended for the clinicians and basic scientists involved in alcohol research.

*Natalia Osna* *Natalia Osna* *Kusum Kharbanda* *Kusum Kharbanda* *Laura Schrum* *Laura Schrum* *Angela Dolganiuc* *Angela Dolganiuc*

